# Supplementary material for: Peroxiredoxins Play an Important Role in the Regulation of Immunity and Aging in Drosophila
Source: Antioxidants (Basel). 2023 Aug 15;12(8):1616. doi: 10.3390/antiox12081616 (PMC10451867; doi:10.3390/antiox12081616)
Supplement: Supplementary file 1 [file antioxidants-12-01616-s001.zip › antioxidants-2516360-supplementary.pdf]

Supplementary material

Figure S1. Immunoblot analysis of dPrx3 and dPrx5 proteins in flies under-expressing dPrx3 specifically in fat bodies of *dprx5* null mutant flies.
Fat bodies were extracted from 10 female flies as described in Krupp's paper [1]. Proteins were extracted from fat bodies of Control and S106 DM flies (Column 1 and 2), and whole body (Column 3) of *y w* flies. Anti-actin antibodies were used for loading control. There was significant under-expression of dPrx3 in fat bodies of S106 DM flies compared to S106 control flies. There wasn't any detectable level of dPrx5 in both S106-Control and DM flies. Since we observed lower level of actin protein in fat bodies compared to the whole body (right), we also provided Coomassie blue staining in order to demonstrate the equal protein loading (left). The abbreviation and genotypes of flies are described in Table 1

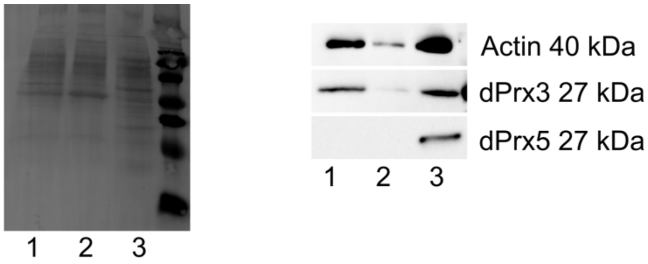

1 - protein extract from fat bodies isolated from Control flies (S106/+; RNAi-dPrx3, prx5/prx5 Ethanol)
2 - protein extract from fat bodies isolated from Experimental flies (S106/+; RNAi-dPrx3, prx5/prx5 MF100)
3 - protein exptract from whole bodies isolated from yw flies

Figure S2. Verification of NP1 driver using UAS-RFP reporter transgene by fluorescent microscopy analysis.

A, Representative images show red fluorescence in the intestinal epithelium of NP1-GAL4/UAS-RFP experimental flies (NP1-RFP) but no significant fluorescence in control flies carrying only UAS-RFP transgene (Control-RFP). No significant fluorescence was observed in other tissues examined.

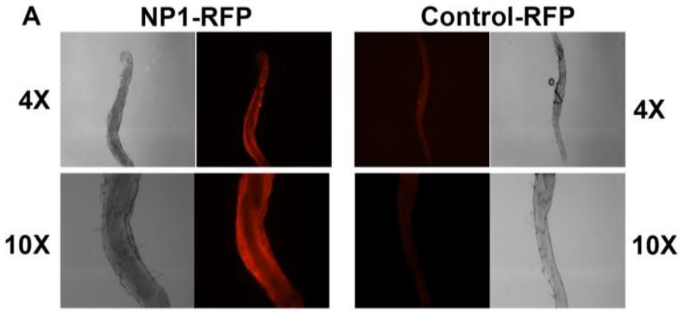

Figure S3. Images of flies with the "smurf" fly phenotype.

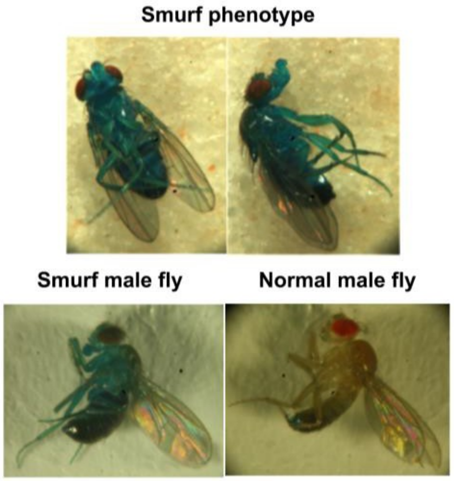

Figure S4. Biological replicate of the experiment depicted in Figure 2. Percentage of dead and 'smurf' flies were scaled to physiological age, or percentage of life span.

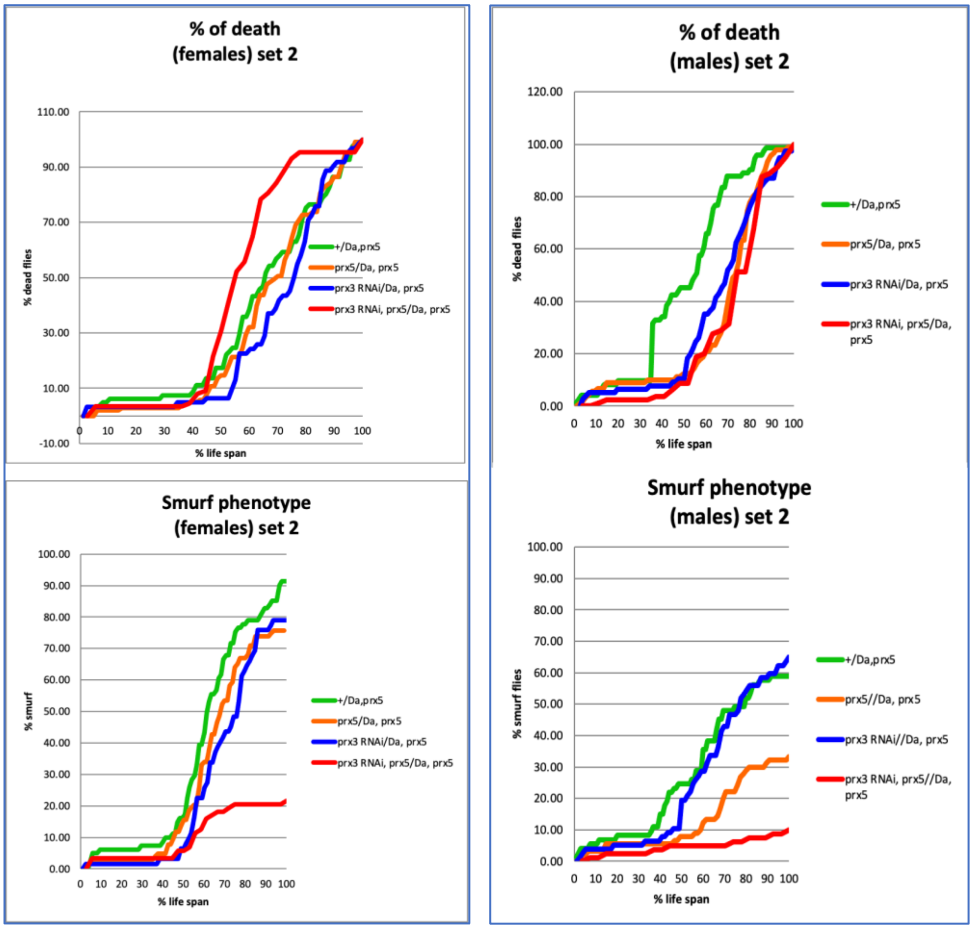

Figure S5. Biological replicate of the experiment depicted in Figure 3. Percentage of dead and 'smurf' flies were scaled to chronological age (A, B) and normalized to percentage of life span (C, D). Genotypes of flies are described in Table 1.

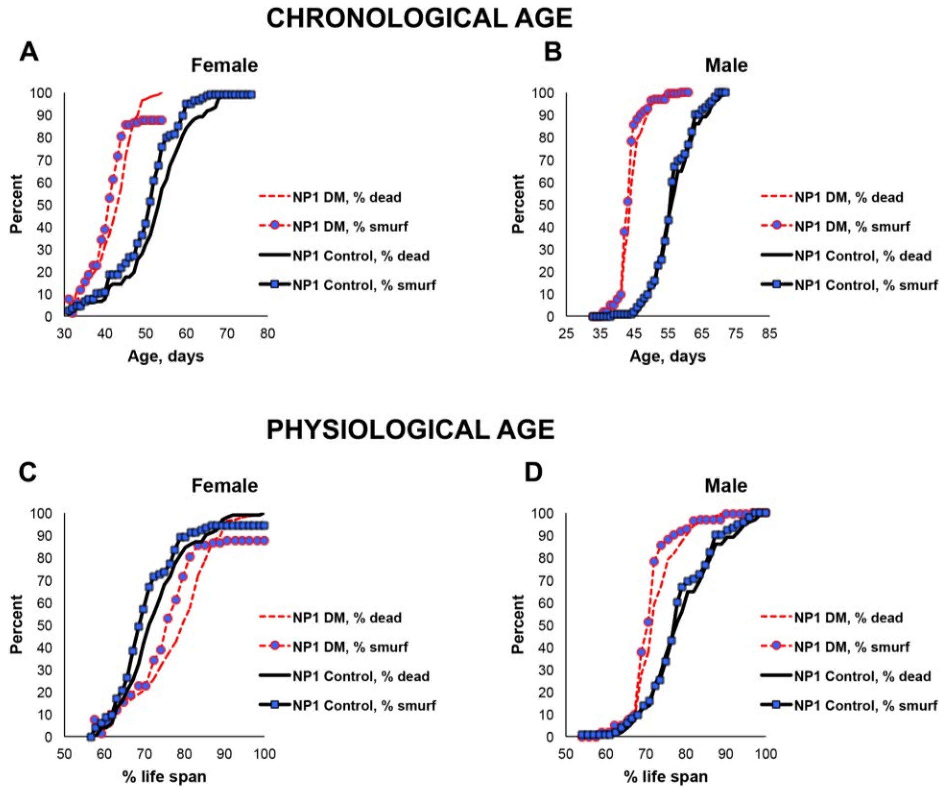

Figure S6. Biological replicate of the experiment depicted in Figure 4C. Effects of antibiotics on life span of the double mutant. In each experiment, approximately 100-125 flies were used for each fly line. The double mutants were kept on the standard food (DMR) and food supplemented with tetracycline (DMT), a combination of ampicillin and chloramphenicol (DMA), or a combination of doxycycline and gentamicin (DMD). The log rank test didn't show significant difference between fly lines.

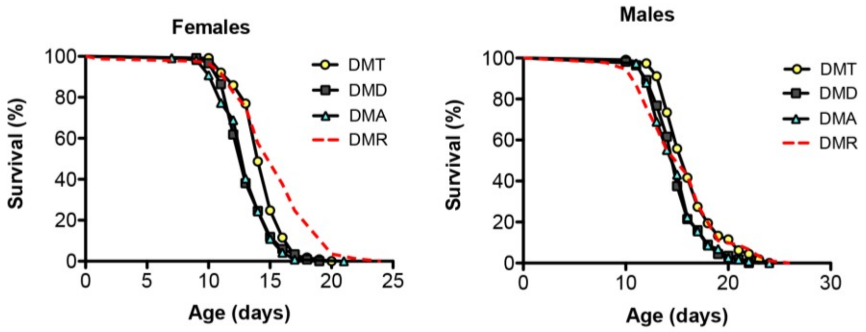

Table S1. The mean life span of the double-mutant flies in response to antibiotics shown in Figure 4C.

Females				Males			
	1	*2*	*3*		*4*	*5*	*6*
**Line**	**Mean, days**	**% vs DM**	**p-value**	**Line**	**Mean, days**	**% vs DM**	**p-value**
**DM-T**	14	-12.5	>0.05	**DM-T**	16	0.0	>0.05
	15	-11.8	>0.05		16	0.0	>0.05
**DM-DG**	13	-18.8	>0.05	**DM-DG**	15	-6.3	>0.05
	13	-23.5	>0.05		14	-12.5	>0.05
**DM-AC**	13	-18.8	>0.05	**DM-AC**	15	-6.3	>0.05
	13	-23.5	>0.05		14	-12.5	>0.05
**DM**	16			**DM**	16		
	17				16		

Columns 1 and 4 indicate the mean life spans observed in two independent biological experiments. Columns 2 and 5 display the percentage changes in the treated groups compared to the DM. Columns 3 and 6 indicate the significance probabilities obtained from the log-rank tests, which were used to compare the survival curve. There were no statistically significant differences between the treated and control groups.

Table S2. Ages of flies collected for the expression of Dipt and AttAB depicted in Figure 6B,C. Genotypes of fly lines are shown in Table 1.

Line	Age	% of Life Span
**DM**	4<br>6<br>12<br>16	15<br>24<br>48<br>64
**TM**	4<br>12<br>15	21<br>51<br>72
**DM Control**	3<br>11<br>24<br>33<br>53	5<br>15<br>38<br>50<br>70
**TM Control**	12<br>29<br>53	15<br>37<br>66

Figure S7. A, Immunoblot analysis of the triple mutant (TM) flies under-expressing dPrx4 in the double mutant *dprx3,dprx5* (DM) background. B, Survivorship curves of DM and TM flies.

A

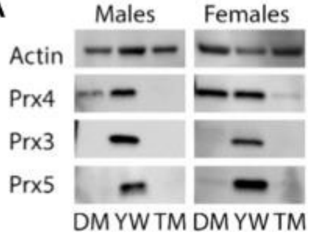

B

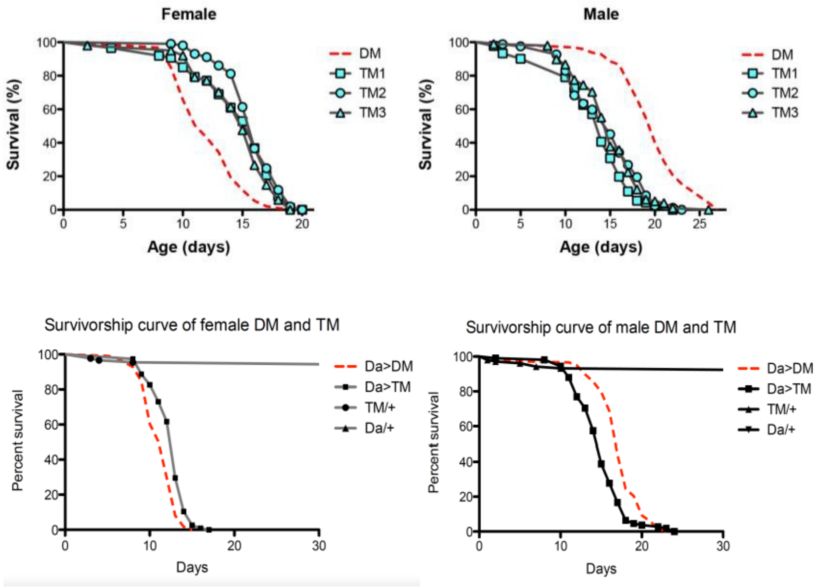

A, The genotypes of fly lines are shown in Table 1. Immunoblots were performed using anti-dPrx5, anti-dPrx3, and anti-dPrx4 antibodies as specified in Materials and Methods. Anti-actin antibodies were used as a loading control. **B**, Biological replicates of experiment depicted in Fig. 6A. In each experiment, approximately 100-125 flies were used for each line. Statistically significant differences ($p<0.05$) between survivorship curves were determined by the log rank test. All statistical data are shown in Table 3 The genotypes of flies are described in Table 1.

1. Krupp, J.J.; Levine, J.D. Dissection of oenocytes from adult Drosophila melanogaster. *J Vis Exp* **2010**, 10.3791/2242, doi:10.3791/2242.
